# Insights into the Roles of B Cells in Patients with Sepsis

**DOI:** 10.1155/2023/7408967

**Published:** 2023-04-22

**Authors:** Xijie Dong, Hao Tu, Shuang Qin, Xiangjun Bai, Fan Yang, Zhanfei Li

**Affiliations:** ^1^Trauma Center/Department of Emergency and Traumatic Surgery, Tongji Hospital of Tongji Medical College, Huazhong University of Science and Technology, Wuhan 430030, China; ^2^Department of Radiation Oncology, Hubei Cancer Hospital, Tongji Medical College, Huazhong University of Science and Technology, Wuhan, China

## Abstract

Sepsis is a life-threatening yet common disease, still posing high mortality worldwide. Sepsis-related deaths primarily occur during immunosuppression; the disease can hamper the numbers and function of B cells, which mediate innate and adaptive immune responses to maintain immune homeostasis. Dysfunction of B cells, along with aggravated immunosuppression, are closely related to poor prognosis. However, B cells in patients with sepsis have garnered little attention. This article focuses on the significance of B-cell subsets, including regulatory B cells, in sepsis and how the counts and function of circulating B cells are affected in patients with sepsis. Finally, potential B-cell-related immunotherapies for sepsis are explored.

## 1. Introduction

Sepsis is a complex disease. Although its etiology has been most recently defined as “a dysregulated host response to infection” [1], different patients may have prior exposure to different pathological processes, such as trauma, burns, or acute abdomen [2]. Also, the pathogens that cause such infections may vary, such as bacteria, viruses, or fungi [3]. Historically adopted etiological therapies primarily focused on the removal or drainage of the infectious foci and the use of antibiotics. Current approaches also pay attention to helping patients recover normal immune function [4, 5].

Patients with sepsis experience immune disorders that can be divided into two processes: pro-inflammatory response and immunosuppression, which occur sequentially or simultaneously [6]. Advances in medical care have helped most patients with sepsis survive pro-inflammatory outbreaks; deaths in patients with sepsis occur mainly during immunosuppression [7, 8]. Immunosuppression is an important cause of late death in early survivors of sepsis and remains difficult to treat [9, 10]. Many studies have shown that lymphocyte failure contributes to immunosuppression in sepsis [2, 4, 10].

B cells are an important subset of lymphocytes that mediate adaptive and innate immune responses by producing antibodies, presenting antigens, and secreting cytokines [11, 12]. They are also able to interact with other innate and adaptive immune cells and affect each other's function during sepsis and inflammation [13–15]. Although B cells are the foundation of immune homeostasis, and B-cell failure will inevitably trigger or exacerbate immunosuppression [4, 16], most studies have focused on how other immune cells change during sepsis while neglecting B cells. B cells play a more important role in sepsis than previously thought [17]; for example, both pathogen clearance and survival were reduced in B-cell-deficient septic mice, and supplemental B cells improved the survival rates of Rag1-deficient mice [18]. Other studies conducted in patients with sepsis or septic shock have associated peripheral blood B-cell depletion and decreased serum IgM levels with poor prognosis. Relieving B-cell depletion and alleviating the decrease of IgM levels likely reduce immunosuppression and improve patient outcomes [19–21]. Clarifying the mechanisms of B-cell immunity in sepsis is prerequisite to developing immunotherapies that can successfully treat immunosuppression.

## 2. The Development and Activation of B Cells

Bone marrow serves as the primary location for B-cell growth and storage. Here, hematopoietic stem cells give rise to diverse B-cell lineages, which then move through various developmental phases, such as pro-B cells, pre-B cells, and immature B cells [22–24]. Most immature B cells leave the bone marrow after their heavy and light-chain immunoglobulin (germline) genes rearrange to form the fully functional B-cell receptor (BCR) and completely differentiate in the periphery to form transitional B cells [24, 25]. Most of these cells are self-tolerant, with few autoreactive cells that undergo clonal deletion or receptor editing in the periphery and eventually develop into naïve B cells [24, 26, 27]. Naïve B cells circulate in the peripheral blood and lymphoid tissues, continue to activate and differentiate when they encounter a homologous antigen, and die within a few days if they do not encounter the homologous antigen [28].

**Figure 1:**
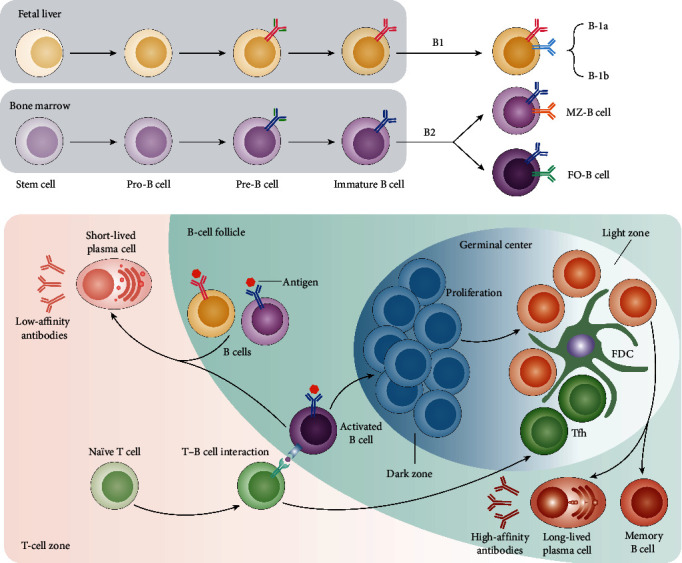
Development and differentiation of B-cell lineage. B cells originate from progenitors that derive from stem cells and undergo multiple developmental stages, with the B1 subset from fetal liver and the B2 subset from bone marrow. The B1 subset is composed of B-1a and B-1b subsets, and the B2 subset is comprised of marginal zone (MZ) and follicular (FO) B cells. The B1 subset and MZ B cells belong to the innate B cells that can be activated independently of T cells and differentiate rapidly into short-lived plasmablasts, which produce low-affinity antibodies without entering the germinal center (GC). On the other hand, FO B cells can interact with activated naive T cells at the T–B border. If the interaction is insufficient, activated B cells differentiate into short-lived plasmablasts that produce low-affinity antibodies. However, if the interaction is sufficient, they can also differentiate into GC B cells. GCs are composed of dark and light zones, and high-affinity clones eventually exit the GCs and differentiate into memory B cells and long-lived plasma cells that secrete high-affinity antibodies. Tfh, follicular helper T. FDC, follicular dendritic cell.

Naïve B cells can be divided into two groups: B1 and B2 cells (Figure 1). The former consists of B-1a and B-1b subsets, and the latter consists of marginal zone (MZ) and follicular (FO) B cells [29]. Mature B cells migrate to peripheral lymphoid tissue and can become active when encountering homologous antigens in a sepsis setting. Their activation response can be divided into two distinct pathways that occur in the absence or presence of T-cell help, respectively.

In general, B1 and MZ B cells are activated independently of helper T cell (Th) help. They can rapidly differentiate into short-lived plasmablasts without entering the germinal center (GC) to quickly build the body's first line of defense against infection before FO B cells play a role [30–33]. In response to bacterial components, MZ B cells exhibit a high sensitivity and are capable of producing antibodies swiftly and sustainably [34]. Further, they can secrete IL-6, as well as CXCL1/CXCL2, within 3–4 hr following *Staphylococcus aureus* infection, contributing to the recruitment of neutrophils to the site of infection, thereby facilitating the speedy elimination of invading pathogens [35]. B-1a cells, on the other hand, are noted for secreting natural IgM and IgG3 to neutralize endotoxins, thereby playing a crucial role in purging pathogenic substances from circulation and improving sepsis [36].

FO B cells become active when the BCR recognizes antigens and key helper signals from antigen-specific Th cells. The antigen binds to the BCR and triggers the necessary gene expression program in the cell, which then leads to T–B-cell interactions through the expression of multiple chemokine receptors and corresponding ligands. If the interaction is insufficient, activated B cells do not enter the GC but develop into extrafollicular plasma cells, producing mainly IgM immunoglobulins that coordinate innate immunity and initiate GC responses. If the interaction is sufficient, the activated B cells return to lymphatic follicles for rapid cloning and expansion to form GC (Figure 1). Centroblasts divide to form centrocytes, migrate outward from the dark zone to form the light zone, interact with dendritic cells and follicular helper T (Tfh) cells, then differentiate into memory B cells and plasmablasts [30, 37–40]. In septic mice, Tfh cells have been shown to help prevent the depletion of FO B cells [41].

## 3. The Significance of B Cells in Sepsis

FO B cells, conventional B cells in the adaptive immune system, are the most numerous of all B-cell lineages. Although FO B cells also participate in T-cell-independent (TI) IgM responses, they are mainly differentiated with the help of T lymphocytes into long-lived plasma cells and class-switched memory B cells that produce high-affinity IgG antibodies and mediate the classical humoral immune response [30]. Long-lived plasma cells consistently produce high-affinity antibodies, and although they do not express antigen receptors and cannot sense antigens, they continue to release their products as high-affinity antibody factories as the first line of defense against reinfection [42–44]. Class-switched memory B cells express specific antigen receptors that trigger a memory response when the antigen is encountered again. During reinfection, memory B cells rapidly differentiate and continue to supply high-affinity antibodies to the blood at the level of existing antibodies, effectively preventing antigen challenge [45]. Secondary infections are less likely to occur due to immune memory or even at all in healthy individuals [44]. However, many memory B cells are depleted in patients with sepsis, hindering humoral immunity and therefore increasing the risk of secondary infection. Indeed, depletion of memory B cells and low antibody levels are closely related to poor patient outcomes, suggesting that an intact humoral immune response is important in sepsis [46–49].

The onset of the FO B-cell-mediated adaptive immune response develops at least 5–7 days after the initial infection. This delay can be fatal if blood or mucosal infection occurs before this time. B1 cells and MZ B cells compensate for this limitation, as they mediate an innate immune response by producing IgM, IgG, and IgA antibodies through a faster TI pathway [32, 50]. B1 cells can not only respond to some TI antigens but also recognize the surface antigens of some Gram-negative bacteria. They can either manage infection by secreting antibodies mainly composed of low-affinity IgM or govern intestinal defense against pathogens by secreting IgA. Moreover, B1 cells can enter the GC for class switching and somatic hypermutation when self-regulation is disordered and produce high-affinity IgG antibodies [51–53]. Remarkably, B-1a cells have reduced excessive inflammation during sepsis by producing large amounts of IL-10 and granulocyte-macrophage colony-stimulating factor in a mouse model of sepsis [54–56]. Studies have also shown that mice with bacterial sepsis have significantly fewer B-1a cells, and B-1a cell-deficient mice are more prone to infection and death. Supplemental B-1a cells can significantly reduce systemic inflammation and improve the survival rates of septic mice [57].

Like B1 cells, MZ B cells can quickly respond to some TI antigens, secrete the first wave of innate immune response antibodies, and modulate the function of T cells and dendritic cells by secreting various cytokines. MZ B cells help regulate rapid systemic anti-infective immunity in host defense responses, dominating the early stages of pathogen eradication [58, 59]. Mice lacking MZ B cells produce fewer antibodies in the early stages after pathogens invade circulation [60, 61]. In addition, MZ B cells can connect the innate immune system and adaptive immune system by initiating low-affinity antibody responses, and they cooperate with lymphocytes and antigen-presenting cells in T-cell-dependent and TI immune responses [62, 63]. Of note, the role of MZ B cells in the inflammatory response remains controversial [64, 65]. On the one hand, MZ B cells can secrete pro-inflammatory cytokines such as IL-6 and C-C motif chemokine ligand 2, which exacerbate the inflammatory response [66]. Pro-inflammatory cytokines produced by MZ B cells can aggravate lipopolysaccharide (LPS)-induced systemic inflammatory responses, and mice lacking MZ B cells are resistant to LPS-induced endotoxic shock [64]. On the other hand, MZ B cells may suppress inflammatory responses during the early stages of infection by producing IL-6, which inhibits the production of tumor necrosis factor-*α* and, therefore, the systemic inflammatory response [67]. MZ B cells are also the cells with the strongest ability to produce inflammatory inhibitory factor IL-10 in vitro [68].

Collectively, the adaptive and innate immune functions of B cells play an important role in sepsis, and the two aspects are closely related. While the abundance of high-affinity antibodies secreted during the adaptive immune response is essential to fighting infection, the role of the innate immune response should not be underestimated [22]. It is well established that sepsis can cause abnormalities in the counts and functions of B cells [16]. These abnormalities, in turn, inevitably lead to impaired immune functions mediated by B cells, resulting in an inability to effectively stifle the attack of pathogens, weakening the protective effect on the body and exacerbating the deterioration of sepsis.

## 4. Regulatory B Cells in Sepsis

Regulatory B cells (Bregs) are a specialized subset of B cells with immunomodulatory functions. The precise source of Bregs remains unknown, however, it has been observed that nearly all subsets of B cells can differentiate into Bregs [69]. They play an important role in maintaining immune homeostasis and tolerance by producing anti-inflammatory cytokines and suppressing inflammatory responses [70].

Breg counts are disturbed in sepsis. Umakoshi et al. [71] found the frequency of CD1d^+^CD5^+^ Bregs in the peripheral blood of cecum ligation and puncture mice increased at 6 hr and returned to baseline at 7 days. The emergence of Bregs accompanied by B lymphocytopenia was the first observable event in sepsis, potentially resulting in immunoparalysis. In a mouse model of endotoxic shock, it was observed that the percentage of CD5^+^CD1d^hi^ Bregs decreased. Transfer of CD5^+^CD1d^hi^ Bregs from healthy wild-type (WT) mice offered protection against severe endotoxic shock [72]. In patients with sepsis, the percentage of IL-10-producing Bregs decreased significantly compared to healthy controls, and this decrease was more pronounced in nonsurvivors than in survivors [73]. Moreover, a transient depletion of memory B cells and Bregs from the circulation was observed in experimental endotoxemia in humans [74].

Bregs may exert a protective effect through their anti-inflammatory function in sepsis. It has been noted that the expression of IL-10 in CD5^+^CD1d^hi^ Bregs was remarkably reduced in severe septic shock mice in response to endotoxin. In addition, the adoptive transfer of CD5^+^CD1d^hi^ Bregs from healthy WT mice (but not from IL-10-deficient mice) led to the downregulation of IFN-*γ* secretion in CD4^+^ T cells, protecting against endotoxic shock. This highlights the significant therapeutic potential of IL-10-producing Bregs in mitigating endotoxic shock [72]. Furthermore, research has shown that IL-35-expressing B cells can regulate Th17 cell function and suppress inflammation. Injection of recombinant IL-35 or IL-35þ Bregs into mice has been observed to resolve inflammation by suppressing effector Th1 and Th17 cell responses while simultaneously regulating Treg-cell responses [75, 76].

The changes in Bregs observed in sepsis suggest a potential role for Bregs in the pathogenesis and outcome of sepsis. The reduction and functional impairment of Bregs may contribute to excessive and dysregulated inflammation in sepsis, leading to poor prognosis.

## 5. Alterations of Circulating B-Cell Counts in Patients with Sepsis

Reduced lymphocyte counts are closely associated with immunosuppression in sepsis and the prognosis [21]. The numbers of B cells and their subsets decline in sepsis but remain debatable. For instance, some studies have reported a decrease in the total number of B cells in the peripheral blood [77, 78], but their counts did not significantly change in other cases [46]. Peripheral blood samples are most analyzed in studies on B cells of patients with sepsis.  Table 1 and Table S1 highlight findings from studies on peripheral blood B cells.

Table 1 includes studies that quantified changes in peripheral blood B-cell counts in patients with sepsis. Two meta-analyses reported changes in the overall count of circulating B cells within 24 hr of onset, but their results did not exactly match because different studies were included [19, 20]. Both reported that circulating B-cell counts in dead patients were significantly lower than in surviving patients, suggesting that B-cell counts are closely related to patient prognosis, which was confirmed in other studies [16, 48]. However, comparisons of patients with healthy controls were inconsistent: one meta-analysis showed no significant difference in circulating B-cell counts between patients and controls [19], while the other reported significantly fewer B cells in patients than in controls [20]. The latter was supported by three other studies [16, 79, 80], and similar results were obtained at other time points (48 hr after admission and days 3, 4, 7, and 8 after onset) [16, 77–80]. Ultimately, most studies indicate lower peripheral blood B-cell counts in patients with sepsis, which is closely related to prognosis. An experimental human endotoxemia model also supports this conclusion [74]. Besides, another study found that the splenic B-cell counts of patients with sepsis were significantly reduced compared with traumatic or critically ill patients without sepsis—greater B-cell loss was linked to persistent sepsis [81].

Table S1 includes studies that monitored how the counts or percentage of peripheral blood B-cell subsets change in patients with sepsis. The selected B-cell subsets and their definitions varied across studies. Results differed even for naïve B cells with relatively uniform nomenclature and surface markers: two studies reported a lower percentage of naïve B cells in patients than in controls [46, 80], and one study found no significant difference in percentages between patients and controls [78], and one study reported a higher percentage in patients than in controls [79]. These studies offer some valuable insights despite their inconsistencies. For example, sepsis indeed affects the B-cell compartment, whose different subsets are uniquely affected. Therefore, future studies should address the subsets separately rather than describing B cells as a whole.

B-cell counts decrease due to a decrease in the source, increased exhaustion, or both. Studies have shown that B-cell depletion is not due to impaired bone marrow production [48, 77] but rather apoptosis [4, 79]. The activation of B cells in patients with sepsis is unsustainable and accelerates apoptosis [79, 80], as shown by a mouse model of sepsis [82, 83]. B cells in humanized mice also show characteristic signs of apoptosis observed in human patients with sepsis [84]. Sensitivity to apoptosis may differ across subtypes, and highly sensitive memory B cells may experience more severe apoptosis [85]. The pathways of B-cell apoptosis are also diverse, including endogenous and exogenous apoptosis [86]. Additionally, patients with sepsis have fewer antigen-presenting T cells in their secondary lymphoid organs, which may impair B-cell maturation and deplete B cells [81, 87, 88]. Besides, circulating B-cell counts may also decrease because B cells flock to the site of inflammation during infection.

## 6. Changes of B-Cell Functions in Patients with Sepsis

**Figure 2:**
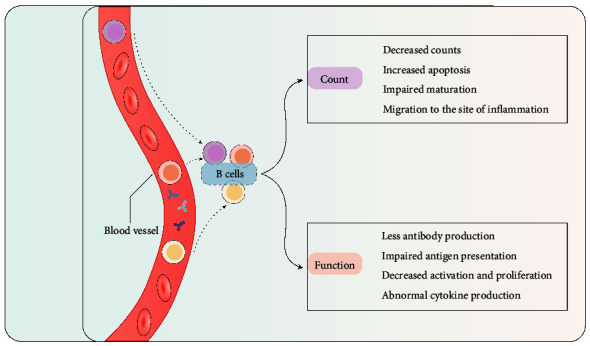
Changes of circulating B cells in patients with sepsis. Significant alterations in both the counts and function of circulating B cells were observed in patients suffering from sepsis.

The B cells in patients with sepsis not only change in numbers but also functions (Figure 2). The main function of B cells is to secrete a variety of antibodies to eliminate pathogens. Patients with sepsis show abnormal serum immunoglobulin levels, which indicate high mortality [89, 90]. Levels of various immunoglobulin subtypes can also change in patients with sepsis. One study found that 16 of 21 patients with septic shock developed hypoimmunoglobulinemia, where levels of IgG and/or IgM decreased [91]. Another study reported that levels of IgG, IgM, and IgA decreased in 61%, 40%, and 4% of 62 patients with septic shock, respectively [89]. This means the level of any immunoglobulin subtype decreases in a patient-specific way, and not every type will decrease in a single patient. Overall levels of IgM decreased in the sepsis patient group when compared to healthy controls, whereas the IgG and IgA levels do not significantly differ. This result has been further verified in vitro [16, 47]. Decreased IgM levels were also found in a larger study that included 332 patients. Here, IgM levels were significantly lower in patients with sepsis or septic shock compared with healthy controls and were significantly higher in survivors than in nonsurvivors [92]. Furthermore, two meta-analyses invariably reported decreased IgM levels within 24 hr in patients with sepsis, which was associated with reduced survival [19, 20]. Overall, decreased levels of IgM are widely implicated in patients with sepsis and poor prognosis.

CD27^+^ B cells are the main antibody-secreting cells, and CD27^+^CD21^hi^ B cells have a stronger IgM-producing capacity than CD27^+^CD21^lo^ B cells [93, 94]. Our recent study found no significant difference in the expression levels of IgM on the surface of B-cell subsets between patients with septic shock and healthy controls, indicating that IgM levels decreased not because of immunoglobulin class switching. Moreover, the IgM levels positively correlated with the number of CD27^+^CD21^hi^ resting memory B cells in patients with septic shock [16]. Therefore, their decrease could be attributed to the depletion of resting memory B cells. Impaired immunoglobulin assembly may also contribute [95]: the heavy and light chains of immunoglobulins are produced and assembled in B cells or plasma cells, and when more light chains than heavy chains are produced intracellularly, the excess light chains are released into the blood. Abnormally elevated concentrations of circulating light chains (free *κ* and *λ* chains) have been observed in patients with sepsis, suggesting impaired immunoglobulin assembly [96, 97]. Furthermore, any given immunoglobulin normally has either two light chains *κ* or *λ*: mature B cells produce only one class of Ig light chains [98, 99]. However, B cells with both light-chain *κ* and *λ* emerged in the blood of patients with sepsis, and higher numbers of B cells expressing both light-chain *κ* and *λ* were positively correlated with free light-chain *κ* and *λ* levels [95].

Several other B-cell functions change in sepsis. For example, during sepsis, the expression levels of human leukocyte antigen-DR decrease in B cells, and the expression levels of PD-1, PD-L1, CD95, and CD80 increase. These phenotypic changes may not only impair the ability of B cells to present antigens but also affect prognosis [80, 100, 101]. Furthermore, the activation and proliferation of circulating B cells in patients with sepsis decrease and tend to failure [102, 103]. Besides, B cells in septic mice abnormally secrete cytokines, resulting in insufficient innate immune response and increased mortality [18]. Overall, these multifaceted changes in B-cell functions do not occur independently and likely affect each other. Altered functions may further deplete B-cell counts and vice-versa to promote immunosuppression in patients with sepsis.

## 7. B-Cell-Related Immunotherapy in Sepsis

B-cell-related immunotherapies for sepsis focus on replenishing the numbers and functions of B cells. Apoptosis exhausts many immune cells, including B cells, and likely contributes to sepsis-induced immunosuppression, and its extent relates to the severity and prognosis [104, 105]. As mentioned earlier, B cells in sepsis show increased expression of CD95 and PD-1. Down-regulating their expression or blocking their binding to ligands may improve the survival rate of septic mice [106–110]. Another strategy includes treatment with anti-CD40, IL-15, or IL-30 to upregulate the expression of antiapoptotic protein Bcl-2 in B cells [109, 111–113]. Caspase-3 is a key enzyme whose activation is a prerequisite to endogenous and exogenous apoptotic pathways; inhibition of its activity has been shown to enhance the survival of septic mice [114–116]. Besides, B cells can also be recovered in septic mice by inhibiting caspase-1, a protein that mediates pyroptosis. Therefore, inhibiting pyroptosis may also be promising [117–119]. Besides, hematopoietic stem cell (HSC) rejuvenation therapies, such as FOXOs and CASIN, could contribute to the regulation of cell growth and survival, increase the pools of common lymphoid progenitor cells, and restore the B-cell counts in the body. This indicates that similar therapies and drugs for HSC rejuvenation could be a promising strategy for restoring B-cell loss [120, 121]. Moreover, animal studies have shown that supplementing specific B-cell subsets through immunotherapy, such as introducing B-1a cells or CD5^+^CD1d^hi^ Bregs, can efficiently reduce inflammatory reactions and prevent organ damage. These results suggest that immunotherapy to reverse B-cell depletion can reduce the harm caused by sepsis [55, 57].

Immunoglobulin supplementation can compensate for the reduced secretion of antibodies by B cells in patients with sepsis. IgG is the main antibody component in serum and body fluids, accounting for about 80% of the total serum immunoglobulin. Although intravenous IgG-only polyclonal immunoglobulin has been suggested to treat sepsis, it has not been proven beneficial [119, 122], even in a large study (*n* = 624) [123]. Polyclonal immunoglobulins could also be supplemented because they are rich in IgM and IgA (IgGAM). A meta-analysis of 19 trials involving more than 1,500 patients with sepsis evaluated the efficacy of IgGAM and found that it significantly reduced mortality [124]. IgGAM therapy has also improved outcomes of patients with sepsis across studies [125, 126]. However, the efficacy of IgGAM must be further verified in larger clinical studies.

It is important to be vigilant about the impact of “sepsis heterogeneity” during immunotherapy. As previously mentioned, patients may have been exposed to different pathogenic factors or infected with various microorganisms. Additionally, they may have varying degrees of severity, be at different disease stages, or have different comorbidities. These variables make immunotherapy challenging. Therefore, personalized treatment should be prioritized when conducting immunotherapy.

## 8. Conclusions

B cells primarily arise in the bone marrow and mature in the periphery. They differentiate into antibody-secreting cells and memory B cells when they encounter antigens. The B1, MZ, and FO B-cell subsets play vital roles in anti-infective immunity and therefore sepsis as well. Bregs have immunomodulatory functions and may play a protective role in sepsis. Most studies report depleted numbers of circulating B cells in patients with sepsis who suffer from poorer prognoses. Different subsets of B cells are not consistently affected across patients, though their general depletion could be to activation-related apoptosis and maturation disorders. Sepsis also impairs B-cell functions. Decreased serum IgM levels are a hallmark of sepsis and poor prognosis. B cells manifest abnormal functions in several ways that could relate to their abnormal counts, and both factors promote immunosuppression. Current promising B-cell-related immunotherapies include reducing depletion or replenishing B cells as well as supplementing with IgGAM. Future studies should pay more attention to B-cell subsets rather than B cells as a whole because sepsis affects the former inconsistently. Finally, immunotherapies for sepsis should prioritize individualization.

## Figures and Tables

**Table 1:** Changes in circulating B-cell counts in patients with sepsis/septic shock.

Reference	Year	Design/sample size	Blood collection time	Results
[16]	2020	Prospective cohort study; survivors (*n* = 57), nonsurvivors (*n* = 24), healthy controls (*n* = 13)	Days 1, 3, and 7 of septic shock onset.	On days 1, 3, and 7, B-cell counts were lower in patients than in healthy controls; on day 7, it was lower in nonsurvivors than in survivors

[19]	2019	Meta-analysis; seven studies^*∗*^ (1999–2017) were included, and sample sizes ranged from 21 to 181	Within 24 hr of sepsis/septic shock onset	There was no significant difference in B-cell counts between patients and healthy controls, but lower in nonsurvivors than in survivors

[20]	2018	Meta-analysis; seven studies^*∗*^ (1999–2013) were included, and sample sizes ranged from 21 to 291	Within 24 hr of sepsis/septic shock onset	B-cell counts were lower in patients than in healthy controls and were lower in nonsurvivors than in survivors

[48]	2020	Prospective cohort study; survivors (*n* = 23), nonsurvivors (*n* = 17)	Within and after 24 hr of sepsis onset	At both time points, B-cell counts were lower in nonsurvivors than in survivors

[77]	2019	Prospective cohort study; ICU patients (*n* = 105, sepsis (*n* = 52), nonsepsis (*n* = 53)), healthy controls (*n* = 63)	At the time of ICU admission and 48 hr after admission	At both time points, B-cell counts in ICU patients were lower than those in healthy controls; there was a decreasing trend in the sepsis group compared with the nonsepsis group, but there was no significant difference

[78]	2018	Prospective cohort study; patients (*n* = 138)	Day 3 of septic shock onset	B-cell counts in patients were lower than the clinical laboratory age-matched reference value

[79]	2017	Prospective cohort study; patients with septic shock (*n* = 24)	Days 1 and 7 of septic shock onset	At both time points, the B-cell counts in patients with sepsis were lower than the population median

[80]	2020	Prospective cohort study; patients with sepsis (*n* = 10), healthy controls (*n* = 10)	Days 1, 4, and 8 of sepsis onset	At all three time points, the relative percentage of B cells in patients was lower than in healthy controls

^*∗*^The studies involved in meta-analysis are no longer discussed separately. ICU, intensive care unit.

## Supplementary Materials

Supplementary Materials Table S1: Changes in percentages or counts of circulating B-cell subset in patients with sepsis/septic shock.
