# Supplementary material for: Insights into the Roles of B Cells in Patients with Sepsis
Source: J Immunol Res. 2023 Apr 22;2023:7408967. doi: 10.1155/2023/7408967 (PMC10148744; doi:10.1155/2023/7408967)
Supplement: Supplementary Materials — Table S1: Changes in percentages or counts of circulating B-cell subset in patients with sepsis/septic shock. [file 7408967.f1.docx]

eTable 1. Changes in percentages or counts of circulating B cell subset in patients with sepsis/septic shock

| Reference Year | Design/Sample Size/  Blood collection time | B cell subsets | Results |
| --- | --- | --- | --- |
| [47]  2016 | Prospective cohort study; Patients with sepsis patients (n = 33), healthy controls (n = 44); 72 h and 8-10 days of sepsis onset. | Naïve B cells (CD19^+^CD27^-^IgD^+^) | The percentage was lower in patients than in healthy controls (8-10 days). |
|  |  | Memory B cells (CD19^+^CD27^+^) | The percentages at both time points did not differ between patients and healthy controls. |
|  |  | Exhausted B cells (CD19^+^CD21^-/lo^CD27^-^CD5^+^) | The percentage and counts in patients were higher than in healthy controls (72 h). |
| [80]  2020 | Prospective cohort study; Patients (n = 10), healthy controls (n = 10); Days 1, 4, and 8 of sepsis onset. | Immature/ transitional B cells (CD19^+^CD24^bright^CD38^bright^) | The percentages were not significantly different between patients and healthy controls (three time points). |
|  |  | Naïve B cells (CD19^+^CD20^+^CD27^-^IgD^+^) | The percentages on days 4 and 8 were lower in patients than in healthy controls. |
|  |  | Pre-switched memory B cells (CD19^+^CD20^+^CD27^+^IgD^+^) | The percentages were lower in patients than in healthy controls (three time points). |
|  |  | Post-switched memory B cells (CD19^+^CD20^+^CD27^+^IgD^-^) | The percentage on day 1 was lower in patients than in healthy controls. |
|  |  | Exhausted B cells (CD19^+^CD20^+^CD27^-^CD21^-/lo^) | The percentages did not differ between patients and healthy controls (three time points). |
|  |  | Plasmablasts (CD19^+^CD20^-^CD38^+^) | The percentages were higher in patients than in healthy controls (three time points). |
| [78]  2018 | Prospective cohort study; Patients (n = 138); Day 3 of septic shock onset. | Naïve B cells (CD19^+^CD27^-^IgD^+^) | The percentages were not significantly different between patients and healthy controls. |
|  |  | Unswitched memory (CD19^+^CD27^+^IgD^+^) | The percentages were not significantly different between patients and healthy controls. |
|  |  | Switched memory B cells (CD19^+^CD27^+^IgD^-^) | The percentages were not significantly different between patients and healthy controls. |
|  |  | Plasma cells (CD19^lo^CD38^hi^CD138^hi^) | The percentages were higher in patients than in healthy controls. |
|  |  | Exhausted-like B cells (CD19^+^CD21^lo^CD95^hi^) | The percentages were higher in patients than in healthy controls. |
| [79]  2017 | Prospective cohort study; Patients (n = 24); Days 1 and 7 of septic shock onset. | Transitional B cells (CD19^+^CD27^-^CD24^+^CD38^+^) | The percentages were not significantly different between patients and healthy controls. |
|  |  | Naïve B cells (CD19^+^CD27^‑^IgM^+^) | The percentages were higher in patients than in healthy controls. |
|  |  | IgM memory B cells (CD19^+^CD27^+^IgM^+^) | The percentages were lower in patients than in healthy controls. |
|  |  | IgA memory B cells (CD19^+^CD27^variable^IgA^+^) | The percentages were lower in patients than in healthy controls. |
|  |  | IgG memory B cells (CD19^+^CD27^variable^IgG^+^) | The percentages were lower in patients than in healthy controls. |
|  |  | Plasmablasts (CD19^+^CD27^+++^CD38^+++^) | The percentages were lower in patients than in healthy controls. |
| [48]  2020 | Prospective cohort study; Survivors (n = 23), non-survivors (n = 17); Within and after 24 h of sepsis onset. | Naïve B cells (CD19^+^CD27^-^) | The percentages did not differ between survivors and non-survivors (two time points). |
|  |  | Memory B cells (CD19^+^CD27^+^) | The percentages were higher in survivors than in non-survivors (two time points). |
|  |  | Antibody-secreting cells (CD19^+^CD27^+^CD38^+^) | The percentages were higher in survivors than in non-survivors (two time points). |
| [16]  2020 | Prospective cohort study; Survivors (n = 57), non-survivors (n = 24), healthy controls (n = 13); Days 1, 3, and 7 of septic shock onset. | Immature/transitional B cells (CD19^+^CD10^+^) | The percentages and counts were decremented in healthy controls, survivors, and non-survivors. |
|  |  | Naïve B cells (CD19^+^CD10^-^CD27^-^CD21^hi^) | The percentages were lower in non-survivors than in survivors and healthy controls, and counts were higher in healthy controls than in survivors and non-survivors. |
|  |  | Resting memory B cells (CD19^+^CD10^-^CD27^+^CD21^hi^) | The percentages were lower in patients than in healthy controls, and counts were decremented in healthy controls, survivors, and non-survivors. |
|  |  | Activated memory B cells (CD19^+^CD10^-^CD27^+^CD21^lo^) | The percentages were higher in non-survivors than in survivors and healthy controls, and counts were higher in healthy controls than in survivors and non-survivors. |
|  |  | Tissue-like memory B cells (CD19^+^CD10^-^CD27^-^CD21^lo^) | The percentages increased progressively in healthy controls, survivors, and non-survivors, and the counts did not differ significantly among the three groups. |
| [100]  2013 | Prospective cohort study; Survivors (n = 34); non-survivors (n = 18), healthy controls (n = 36); Days 0, 3, 7, 14, and 28 of septic shock onset. | Activated regulatory B cells (CD19^+^CD23^+^) | The percentages were higher in survivors than in healthy controls, and counts were lower in patients than in healthy controls (all time points). |
|  |  | Early activated B cells (CD19^+^CD69^+^) | The percentages were higher in patients than in the healthy controls, and the counts were not significantly different from the healthy controls (all time points). |
|  |  | B1a cells (CD19^+^CD5^+^) | The percentages were not significantly different among the three groups, and the counts were lower in patients than in healthy controls (all time points). |
